# Treatment results of small bowel perforations due to unusual causes

**DOI:** 10.12669/pjms.344.14808

**Published:** 2018

**Authors:** Mustafa Yener Uzunoglu, Fatih Altintoprak, Enis Dikicier, Ismail Zengin

**Affiliations:** 1 Mustafa Yener Uzunoglu, MD. Department of General Surgery, Sakarya University, Research and Educational Hospital, Sakarya, Turkey; 2 Fatih Altintoprak, MD. Department of General Surgery, Istinye University Faculty of Medicine, Istanbul, Turkey. Department of General Surgery, Sakarya University, Research and Educational Hospital, Sakarya, Turkey; 3 Enis Dikicier, MD. Istinye University Faculty of Medicine, Istanbul, Turkey; 4 Ismail Zengin, MD. Department of General Surgery, Sakarya University, Research and Educational Hospital, Sakarya, Turkey

**Keywords:** Non-traumatic Perforation, Perforation, Small bowel perforation, Bezoar

## Abstract

**Objectives:**

Although non-traumatic Small Bowel Perforations (SBPs) are rare, they have high rates of morbidity and mortality in case of late presentation. Aetiological factors vary across different geographical regions. In this paper, SBPs caused by anything other than trauma and other well-known causes are presented and the current literature is reviewed.

**Methods:**

The study was conducted at General Surgery Clinics of two different tertiary university hospitals between January 2008 and September 2016. The authors directly involved in managing the patients. This study was approved by the ethical institutional board and was performed at the Department of General Surgery, School of Medicine, Sakarya University. The medical records of patients retained in both hospitals are electronic. Medical records of subjects who had undergone emergency operations with a prediagnosis of acute abdomen in single center, and were determined to have SBPs due to unusual causes, were investigated retrospectively. Patients with aetiological factors such as trauma, mesenteric vascular disease, internal and external hernias, intra abdominal adhesions, inflammatory bowel diseases, and iatrogenic causes were excluded.

**Results:**

In total, 35 patients were evaluated, 20 (57.1%) males and 15 (42.9%) females. The mean age of the cases was 51.6 (18–88) years. Mean time until admission at the hospital was 1.4 days (range 0.25–7 days). The most frequent aetiological factors were various malignancies (10 cases, 28.5%) and perforation of Meckel’s diverticulum (8 cases, 22.8%). It was surprising to detect a considerable rate of perforation due to bezoars (6 patients, 17.1%).

**Conclusions:**

Post-operative consequences of SBPs due to unusual causes are similar with those related to common, known causes. Factors affecting the clinical course are presentation time and patients’ clinical status in admission, not aetiology.

## INTRODUCTION

Small Bowel Perforations (SBPs) due to causes other than trauma and known common aetiological factors (mesenteric vascular disease, internal and external hernias, intraabdominal adhesions, inflammatory bowel diseases, and iatrogenic) are also called spontaneous or non-traumatic SBPs.^1^,^2^ The most frequent causes include various malignancies, infections, and non-specific inflammation.^3^ In Western populations, malignancies are the most frequent aetiological factors, whereas infections, and in particular typhoid fever, are the primary causes in developing populations.^3^,^4^ Cases that also show inflammation (as assessed histopathologically) that cannot be linked to a specific disease are deemed to have idiopathic or non-specific inflammation.^4^

Presentation at the hospital is generally late, and patients are in an impaired physical condition due to diffuse peritonitis.^5^ This is why outcomes in the postoperative period are still poor despite developments in surgical-radiologic techniques and intensive care conditions.^1^,^5^ The first-line choice for treatment of this disease is a surgical approach, and timing of the operation is a major factor determining success.^1^

In this paper, we aimed to present the unusual causes of small bowel perforations, treatment results and investigate whether the clinical course is different from other SBPs due to common, well known aetiologies.

## METHODS

The medical records of 224 patients with small bowel perforation were investigated retrospectively; the cases had undergone emergency operations with a pre-diagnosis of acute abdomen in the General Surgery Clinics of two different tertiary university hospitals between January 2008 and September 2016. Cases with causes such as trauma, mesenteric vascular disease, internal or external hernias, intraabdominal adhesions, inflammatory bowel diseases, and iatrogenic perforations were excluded. The remaining 35 cases (15.6 %) were included and evaluated.

The surgeon who was to conduct the operation decided on the surgical procedure, according to the haemodynamic parameters of the patient and signs during the operation. Primary repair or anastomosis following segmentary small bowel resection was generally preferred in patients without intensive intraabdominal problems or abscesses, and in those free of signs of risk factors for intestinal anastomosis in their haemodynamic and laboratory parameters (e.g., acidosis, hypoproteinemia, severe hypotension, chronic steroid intake). Enterostomy, following segmentary small bowel resection, was used in the other patients.

The patients were divided into two groups, according to the presentation time, as follows: those who were admitted within the first 24 hours (Group 1), and those who admitted after 24 hours (Group 2). The two groups were compared statistically regarding postoperative morbidity and mortality. The Student’s t-test was used for statistical evaluations, and p value < 0.05 was considered to indicate statistical significance.

## RESULTS

Of the 35 patients, 20 (57.1%) were males and 15 (42.9%) were females. The mean age was 51.6 years (range; 18–88). All cases had signs of localized or diffuse peritoneal irritation at their first presentation, and all of them came to the hospital with a complaint of abdominal pain (100%). Their complaints at presentation are listed in Table-I.

**Table-I:** Complaints and sign of admission in all patients (n=35, 100%).

Abdominal pain	35(%100)	Abdominal tenderness	35(%100)
Vomiting	26(%74)	Rebaund tenderness	29(%83)
Constipation	11(%31)	Dehydrated appearance	17(%48)
Weight loss	4(%12)	Anemic appearance	3(% 8)
Melanotic stool	2(%6)	Intraabdominal mass	2(%6)
Diarrhea	2(%6)		

Mean time until presentation at the hospital was 1.4 days (range; 0.25–7 days). Time from the onset of complaints to attendance at the hospital was over 24 hour in 11 (31.4%, Group 2) patients and within 24 hour in 24 (68.6%, Group 1) patients.

### Medical history, Laboratory and radiological findings all patients’ as follow: Medical history

Fifteen (42.8%) patients had comorbid diseases, and 10 (28.5%) patients had histories of previous abdominal surgeries. In two (5.7%) patients, the reasons for previous abdominal surgeries were malignancies (one case of small bowel stromal tumour and one of cervical cancer). One (2.8%) patient had a history of long-term steroid intake due to Pemphigus. One patient had been operated on due to volvulus of the stomach five days previously. Details of the comorbid diseases and previous operations are provided in Table-II.

**Table-II:** Co-morbid diseases and aetiologies of previous abdominal surgery.

Co-morbid disease	n=15; %100	Previous abdominal surgery	n=10;%100
Hypertension (HT)	3(%20)	Laparotomy	4(%40)
Coronary arter disease (CAD)	2 (%13)	Peptic ulcer	3(%30)
Cerebrovascular Accident CVA)	1(%7)	Cervix cancer	1(%10)
Pemphigus	1(%7)	Cholecystectomy	1(%10)
Pulmoner tuberculosis	1(%7)	Sb stromal tumor	1(%10)
HT + CAD	2 (%13)		
DM + CVA	1(%7)		

### Laboratory Findings

Leukocytosis in 26 (74.2%) cases (>11,000/mm^3^), leucopenia in one (2.8%) case (3,400/mm^3^), and normal leukocyte values in eight cases (22.8%) (normal range 5200–10800/mm^3^). The patient with leucopenia also had moderate anaemia (Hb 7.8 g/dL).

### Radiological Findings

Intraabdominal free air was present in the abdominal X-rays of 16 (45.7%) patients. In 14 (40%) patients, air-fluid levels were observed, suggestive of intestinal obstructions. In five (14.2%) patients, the abdominal X-rays were normal. In the cases where free air was observed, surgery was decided without additional radiological investigations. Five patients (14.2 %) who had normal abdominal X-rays findings underwent abdominal ultrasonography (USG), and were determined intraabdominal free fluid in all cases. Abdominal Computed Tomography (CT) examination was performed in all cases except those who had intraabdominal free air in the abdominal X-rays. Intraabdominal free fluid and dilated intestines were common signs in all patients who underwent CT investigations. In addition, in four patients (21%), there were images of masses that appeared to originate from the small bowel, and seven patients had bezoars (36.8%)

### Operation Findings

Perforation was determined in a single location in 31 (88.5%) patients; in 3 (8.6%) cases, perforation was seen in two locations, and multiple perforations were detected in 1 (2.8%) case (the patient was operated on for volvulus of the stomach). The jejunum was the perforated location in 10 (28.5%) patients and ileal perforations were present in 25 (71.5%) patients. Primary repair, enterostomy following segmentary small bowel resection, and primary anastomosis following segmentary small bowel resection were performed in 4 (11.4%), 9 (25.7%), and 22 (62.8%) patients, respectively.

No patient underwent a planned re-laparotomy. Five (14.2%) cases underwent re-laparotomy due to early post-operative complications, as follows: reperforation in two (5.7%) patients (patients with intestinal tuberculosis and volvulus of the stomach), intraabdominal abscess in two (5.7%) patients, and anastomosis leak in one (2.8%) patient.

Except for the indications for re-laparotomy, 17 (48.5%) patients had various comorbidities, as follows: infection of the wound location in 8 (22.8%) cases, cardiopulmonary complications in 7 (20%), evisceration in one (2.8%), and enterocutaneous fistula (EFC) in one (2.8%) patient.

### Statistical Analysis

complication rates were higher in subjects who had presented to the hospital after 24 hour (Group 2) compared to those who had presented within the first 24 hour (Group-1) (p = 0.03).

In seven (20%) patients, the treatment process resulted in mortality. Causes of death were as follows: three (7.5%) cases of perforation of Meckel’s diverticulum, two (5%) cases of intestinal tuberculosis, one (2.5%) case of multiple perforations (possibly due to microembolism) due to sepsis in the early post-operative period, during intensive care follow-up, one (2.5%) case of volvulus of the stomach and one (2.5%) case of radiation enteritis due to comorbid cardiovascular diseases. Except for the patient operated on for volvulus of the stomach, all patient deaths occurred during the early post-operative period in those who presented late to the hospital (Group 2) (p = 0.002).

### Aetiological Factors

**Fig.1:**
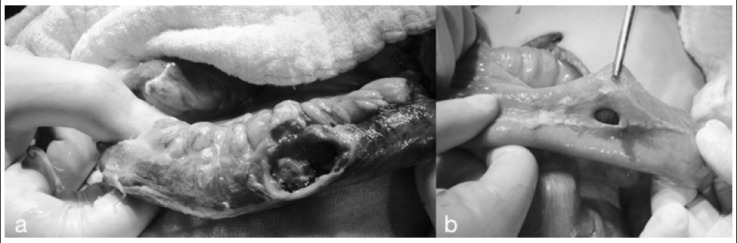
Perforations due to non-specific inflammation: late presentation (after 24 hours); large perforation area with containing excess necrotic tissue (a), early presentation (within first 24 hours); smaller perforation area with no necrotic tissue (b)

**Fig.2:**
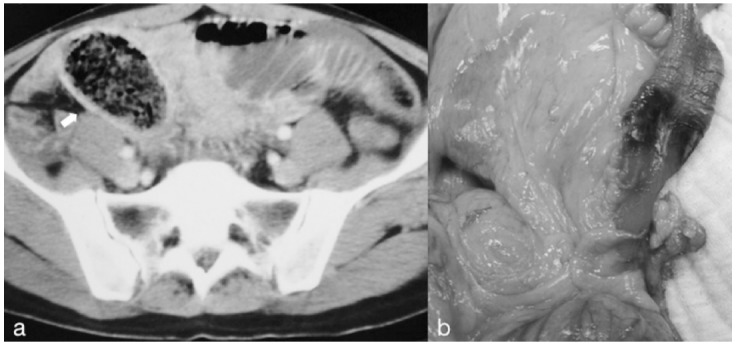
Perforation due to phytobezoar: intraluminal bezoar imaging and inflammation findings in surrounding tissues on CT (a), intraoperatif view; necrotic area and perforation at ileum depending on bezoar pressure (b)

Among the aetiological factors, there were malignancies in 10 (28.5%) patients (lymphoma in 8 cases and stromal tumour of the small bowel in 2 cases). Other causes were as follows: perforation of Meckel’s diverticulum (8 patients, 22.8%), phytobezoar (6 patients, 17.1%), non-specific inflammation (5 patients, 14.2%), intestinal tuberculosis (3 patients, 8.5%), multiple perforations (possibly due to microembolism; one patient, 2.8%), radiation enteritis (1 patient, 2.8%), and a foreign body (fishbone; one patient, 2.8%) (Table-III) (Fig. 1 and 2).

**Table-III:** Aetiologic factors.

Aetiology	n=35 (100, %)
Malignancies	10 (%28,5)
Lymphoma	8 (%22,8)
Stromal tumor	2 (%5,7)
Meckel’s diverticulum	8 (%22,8)
Phytobezoar	6 (%17,1)
Non-spesific inflammation	5 (%14,2)
Tuberculosis	3 (%8,5)
Unknown	1 (%2,8)
Radiation enteritis	1 (%2,8)
Foreign body	1 (%2,8)

## DISCUSSION

There are many well-known aetiological factors that cause SBPs, but some factors are only rarely observed. These are considered rare or unusual aetiological causes, and are sometimes classified in the surgical literature as non-traumatic or spontaneous SBPs. They occur at low rates, and SBPs resulting from these causes can be divided into two groups: those with defined and undefined aetiological factors.^4^,^5^

However, factors considered to be rare vary by geographical region and the socioeconomic status of the case. For example, cases of perforation related to typhoid fever are extremely rare in Western populations, but this is the most frequent cause in Eastern populations.^6^ Similarly, intestinal tuberculosis is extremely rare in developed countries but is still an important intestinal problem in some geographical regions.^7^

If, in Western populations, inflammatory bowel diseases are not taken into consideration, malignancies are more often encountered aetiological factor for SBPs. There are reported cases of SBPs related to primary tumours of the small bowel, such as T- and B-cell lymphomas, stromal tumours, adenocarcinomas, and carcinoids.^8^-^18^ Among these, lymphomas are the first to be kept in mind when considering primary small bowel malignancies resulting in perforations.^13^ In a serial study of 1062 patients at the Mayo Clinic^13^ and a 37-year investigative series of SBP in patients with intestinal lymphoma^14^, the rate of SBP was 9% in GIS lymphomas, the majority due to intestinal metastasis. Turkey is a synthesis of Eastern and Western society in terms of its cultural status and geographic location. This is reflected in our findings. Malignancies were the most frequent causes in our study, similar to Western populations, but we identified factors that are specific to Eastern populations and reflect the social characteristics of our country, although at lower rates.

Meckel’s diverticulum is a well-known congenital state that results in an inflammatory process and perforation due to various factors.^19^,^20^ Marked social or development differences in this condition should not be expected because of its congenital nature. Although Meckel’s diverticulum’s complication rate has been reported low (4%) during lifetime^21^, in the present study, it was the second most frequent aetiological cause.

SBPs due to the ingestion of foreign bodies is a major issue, and there are two types of foreign bodies: those that are ingested accidentally, and those that accumulate in the gastrointestinal system due to chronic consumption. Chicken and fish bones are examples of the first group. Cases of perforation (particularly in the oesophagus) due to chicken bones have been reported globally, whereas most cases due to fish bones are associated with the consumption of large fish from the oceans rather than smaller fish from interior bodies of water, and thus typically occur in countries with ocean coastlines.^4^,^5^ In our study, a case of perforation related to fish bones was a sailor who had been working in international waters and was in Turkey only temporarily.

Phytobezoars are the most common foreign bodies that form in the gastrointestinal system due to the chronic consumption of a given food.^22^ Some facilitating factors for the formation of phytobezoars, and related nutrients, have been defined.^23^ Numerous studies have documented small bowel obstructions caused by the consumption of high amounts of fibre. Turkey is an ideal geographical region for growing persimmon, which is high in fibre, and therefore cases with small bowel obstruction related to phytobezoars are relatively frequent.^22^ Although we are experienced in the pre-operative diagnosis of intestinal obstructions related to phytobezoars, six patients in our study ultimately experienced perforations due to such obstructions. However, these cases presented late to the hospital, and already had bezoars (based on CT images) at presentation.

Nowadays, the therapeutic outcomes of cases with SBPs are better than past decades due to developments in visualisation methods, surgical techniques, and intensive care conditions. However, prognosis are still poor in some patients. The most important reason, confirmed by our results, is related to the timing of presentation at the hospital.^24^-^26^ The high rates of morbidity and mortality are expected and are related to the degree of intraabdominal pollutants and the systemic influences of intraabdominal sepsis.

In conclusion, the clinical course of SBPs due to unusual causes are not different from that of perforations related to common, known causes. Most important issue is surgical intervention before the development of signs of intraabdominal sepsis. Factors affecting of clinical course are presentation time and patients’ clinical status in admission, not aetiology.

### Authors Contribution

**MYU:** Conceived, designed and did statistical analysis & editing of manuscript.

**ED, IZ:** Did data collection and manuscript writing.

**FA:** Did review and final approval of manuscript.

## References

[1] Mastboom WJ, Kuypers HH, Schoots FJ, Wobbes T (1989). Small-bowel perforation complicating the open treatment generalized peritonitis. Arch Surg.

[2] Mischinger HJ, Berger A, Kronberger L, Fellbaum C (1989). Spontaneous small bowel perforation. Acta Chir Scand.

[3] Eid HO, Hefny AF, Joshi S, Abu-Zidan FM (2008). Non-traumatic perforation of the small bowel. Afr Health Sci.

[4] Freeman HJ (2014). Spontaneous free perforation of the small intestine in adults. World J Gastroenterol.

[5] Leijonmarck CE, Fenyö G, Räf L (1984). Nontraumatic perforation of the small intestine. Acta Chir Scand.

[6] Talwar S, Sharma RK, Mittal DK, Prasad P (1997). Typhoid enteric perforation. Aust N Z J Surg.

[7] Rauf A Wani, Fazl Q Parray, Nadeem A Bhat, Mehmood A Wani, Tasaduq H Bhat, Fowzia Farzana (2006). Nontraumatic terminal ileal perforation. World J Emerg Surg.

[8] Mcdermott EWM, Cassidy N, Heffernan Sj (1992). Perforation through undiagnosed small bowel involvement in primary thyroid lymphoma during chemotherapy. Cancer.

[9] Miholic J, Schlappac O, Klepetko W, Kölbi H, Szepesi T, Moeschl P (1987). Surgical therapy of radiation-induced small-bowel lesions. Arch Surg.

[10] Sager GF (1978). Primary malignant tumors of the small intestine. A twenty-two year experience with thirty patients. Am J Surg.

[11] Freeman HJ (2003). Free perforation due to intestinal lymphoma in biopsy-defined or suspected celiac disease. J Clin Gastroenterol.

[12] Kelemen K, Yu QQ, Howard L (2004). Small intestinal angiosarcoma leading to perforation and acute abdomen:a case report and review of the literature. Arch Pathol Lab Med.

[13] Vaidya R, Habermann TM, Donohue JH, Ristow KM, Maurer MJ, Macon WR (2013). Bowel perforation in intestinal lymphoma:incidence and clinical features. Ann Oncol.

[14] Shiraishi M, Hiroyasu S, Nosato E, Shimoji H, Kusano T, Muto Y (1998). Perforation due to metastatic tumors of the ileocecal region. World J Surg.

[15] Verma D, Stroehlein JR (2006). Adenocarcinoma of the small bowel:a 60- yr perspective derived from M.D. Anderson Cancer Center tumor registry. Am J Gastroenterol.

[16] Ise N, Kotanagi H, Morii M, Yasui O, Ito M, Koyama K (2001). Small bowel perforation caused by metastasis from an extra-abdominal malignancy:report of three cases. Surg Today.

[17] Yabuki K, Tamasaki Y, Satoh K, Maekawa T, Matsumoto M (2000). Primary Gastric Lymphoma with Spontaneous Perforation:Report of a Case. Surg Today.

[18] Ohkura Y, Lee S, Kaji D, Ota Y, Haruta S, Takeji Y (2015). Spontaneous perforation of primary gastric malignant lymphoma:a case report and review of the literature. World J Surg Oncol.

[19] Sozen S, Tuna Ö (2012). A rare case of perforated Meckel's diverticulum presenting as a gastrointestinal stromal tumor. Arch Iran Med.

[20] Butler JS, Collins CG, McEntee GP (2010). Perforated jejunal diverticula:a case report. J Med Case Rep.

[21] Ding Y, Zhou Y, Ji Zh, Zhang J, Wang Q (2012). Laparoscopic management of perforated Meckel's diverticulum in adults. Int J Med Sci.

[22] Bingham JR, Causey MW, Haque MI (2014). Phytobezoar with in Meckel's diverticulum:an unusual cause of intestinal obstruction. Am Surg.

[23] Altintoprak F (2010). Gastric outlet syndrome associated with a recurrent trichobezoar:report of a case. Turk J Gastroenterol.

[24] Ben-Baruch D, Powsner E, Wolloch Y, Dintsman M (1990). Free perforation of small intestine in adults. Panminerva Med.

[25] Sefr R, Rotterova P, Konency J (2001). Perforation peritonitis in primary intestinal tuberculosis. Dig Surg.

[26] Akyildiz HY, Akcan AC, Sözüer E, Küçük C, Yilmaz N, Artiş T (2009). Unusual causes of intestinal perforation and their surgical treatment. Ulus Travma Acil Cerrahi Derg.

